# Supplementary material for: Structures of a mammalian TRPM8 in closed state
Source: Nat Commun. 2022 Jun 3;13:3113. doi: 10.1038/s41467-022-30919-y (PMC9166780; doi:10.1038/s41467-022-30919-y)
Supplement: Supplementary file 1 — Supplementary Information [file 41467_2022_30919_MOESM1_ESM.pdf]

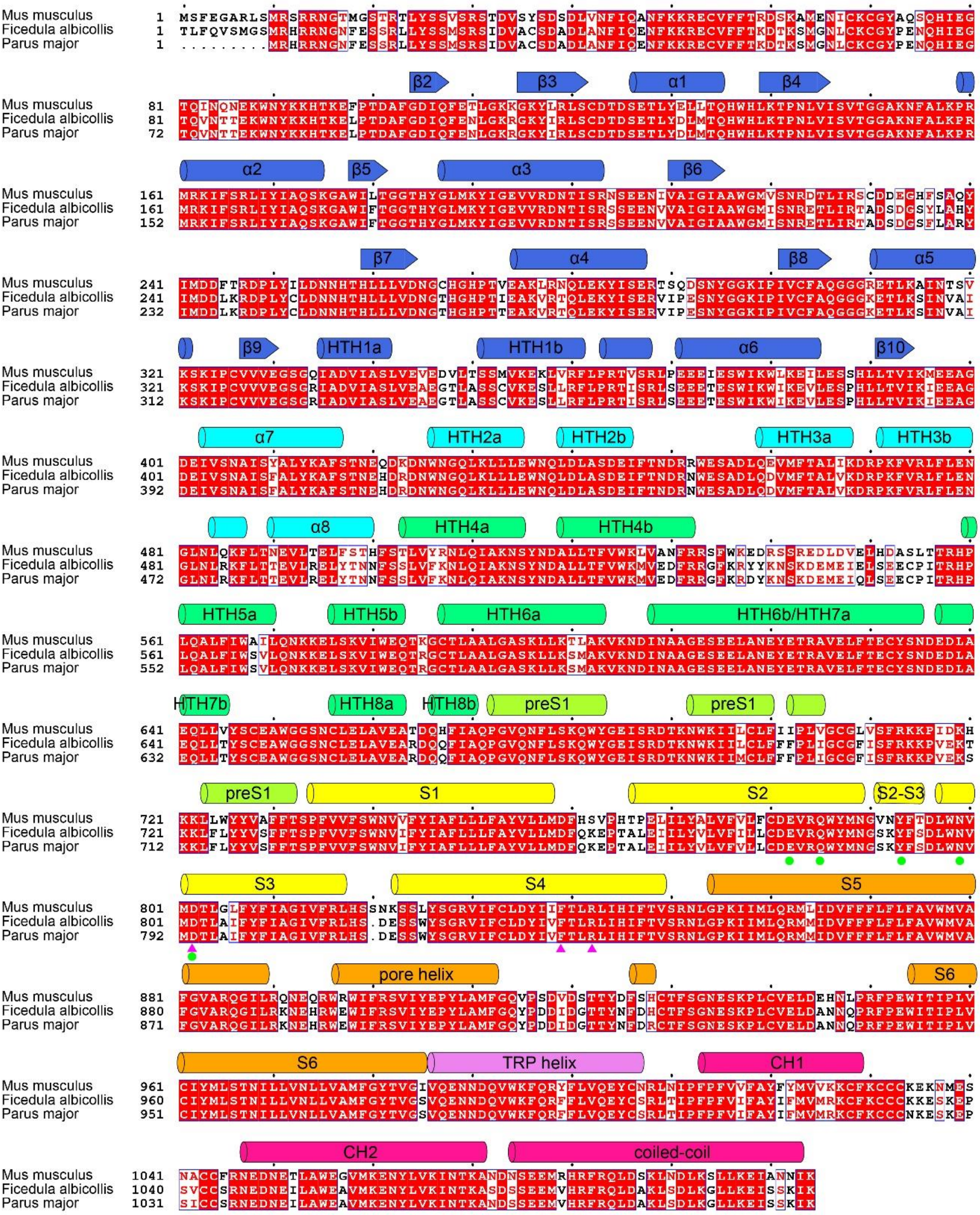

Supplementary Figure 1. Sequence alignment of TRPM8 orthologs. Secondary structures colored as in Figure 2 are indicated by cylinders (helices) and arrows (β-strands). Conserved residues are highlighted in red. Ca^{2+} and icilin binding sites are highlighted with green spheres and magenta triangles, respectively. TRPM8 from both *Ficedula albicollis* (FaTRPM8) and *Parus major* (PmTRPM8) share 82% sequence identity and 91% sequence similarity to mouse TRPM8 (MmTRPM8). HTH, helix-turn-helix; CH, C-terminal helix.

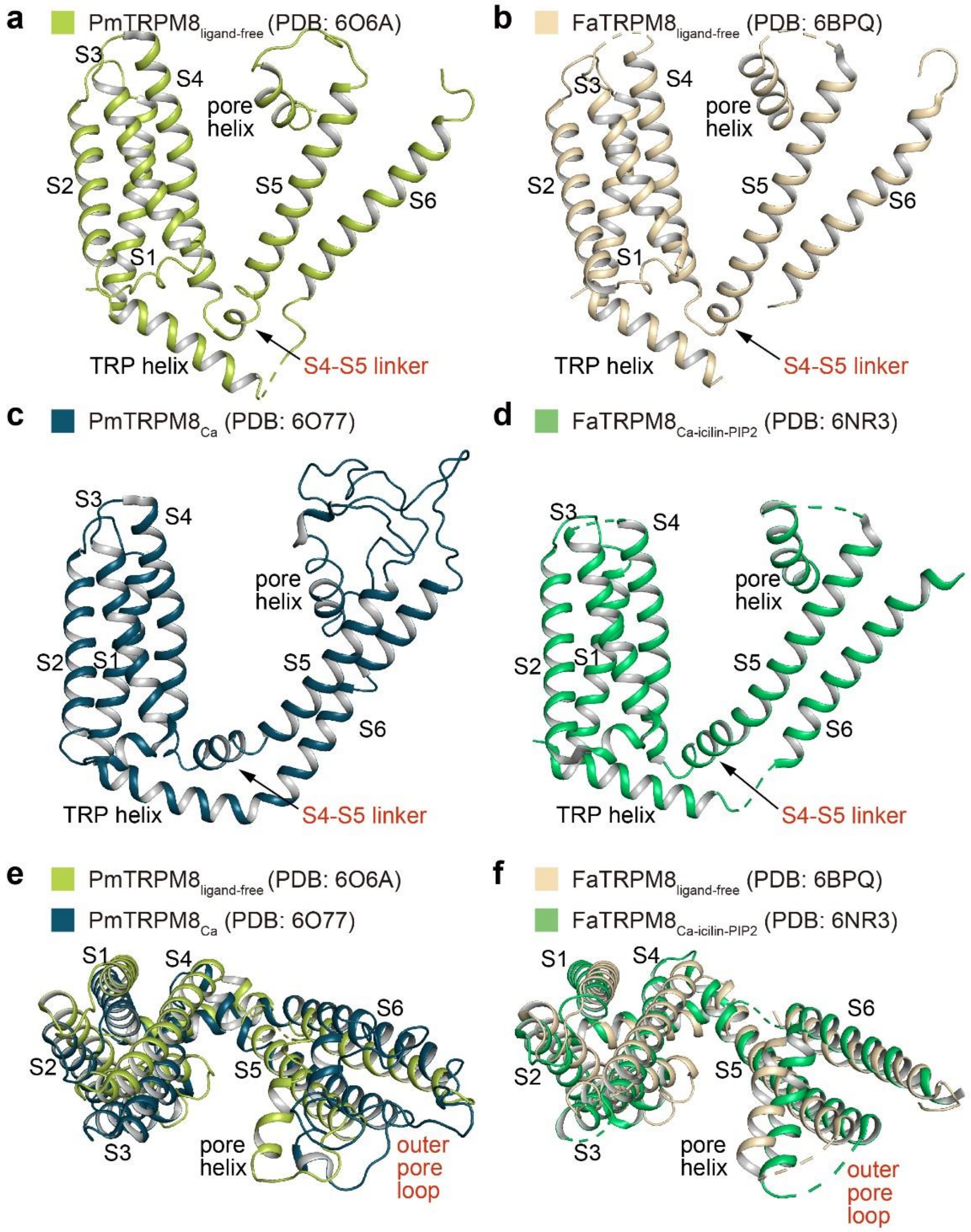

Supplementary Figure 2. Structures of transmembrane domains in published bird TRPM8 structures. a, Side view of S1–S6 transmembrane domain in PmTRPM8$_{\text{ligand-free}}$. For clarity, only one subunit is shown. **b**, Side view of S1–S6 transmembrane domain of FaTRPM8$_{\text{ligand-free}}$. **c**, Side view of S1–S6 transmembrane domain of PmTRPM8$_{\text{Ca}}$. **d**, Side view of S1–S6 transmembrane domain of FaTRPM8$_{\text{Ca-icilin-PIP2}}$. **e**, Top view of structure superimposition of transmembrane domains in PmTRPM8$_{\text{ligand-free}}$ and PmTRPM8$_{\text{Ca}}$ when the entire channels are aligned. **f**, Top view of structure superimposition of transmembrane domains in FaTRPM8$_{\text{ligand-free}}$ and FaTRPM8$_{\text{Ca-icilin-PIP2}}$ when the entire channels are aligned.

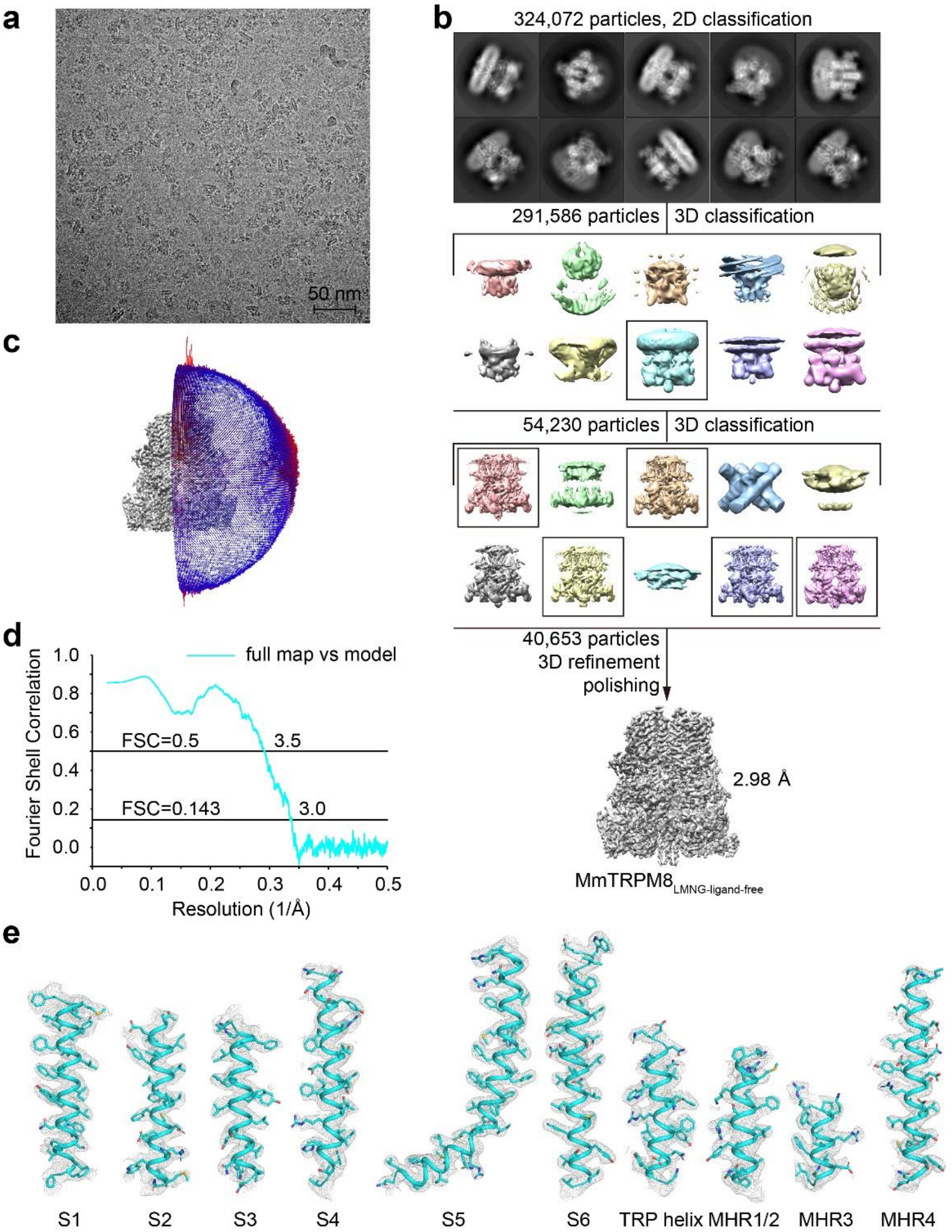

Supplementary Figure 3. Structure determination of MmTRPM8$_{\text{LMNG-ligand-free}}$. **a**, Representative cryo-EM micrograph of MmTRPM8$_{\text{LMNG-ligand-free}}$. **b**, Flowchart of image processing for MmTRPM8$_{\text{LMNG-ligand-free}}$ particles. **c**, Angular distribution plot of particles included in the final *C4*-symmetric 3D reconstruction of MmTRPM8$_{\text{LMNG-ligand-free}}$. **d**, The FSC curves for cross-validation between the map and the model of MmTRPM8$_{\text{LMNG-ligand-free}}$. Source data are provided as a Source Data file. **e**, Sample maps at 10 helices of MmTRPM8$_{\text{LMNG-ligand-free}}$.

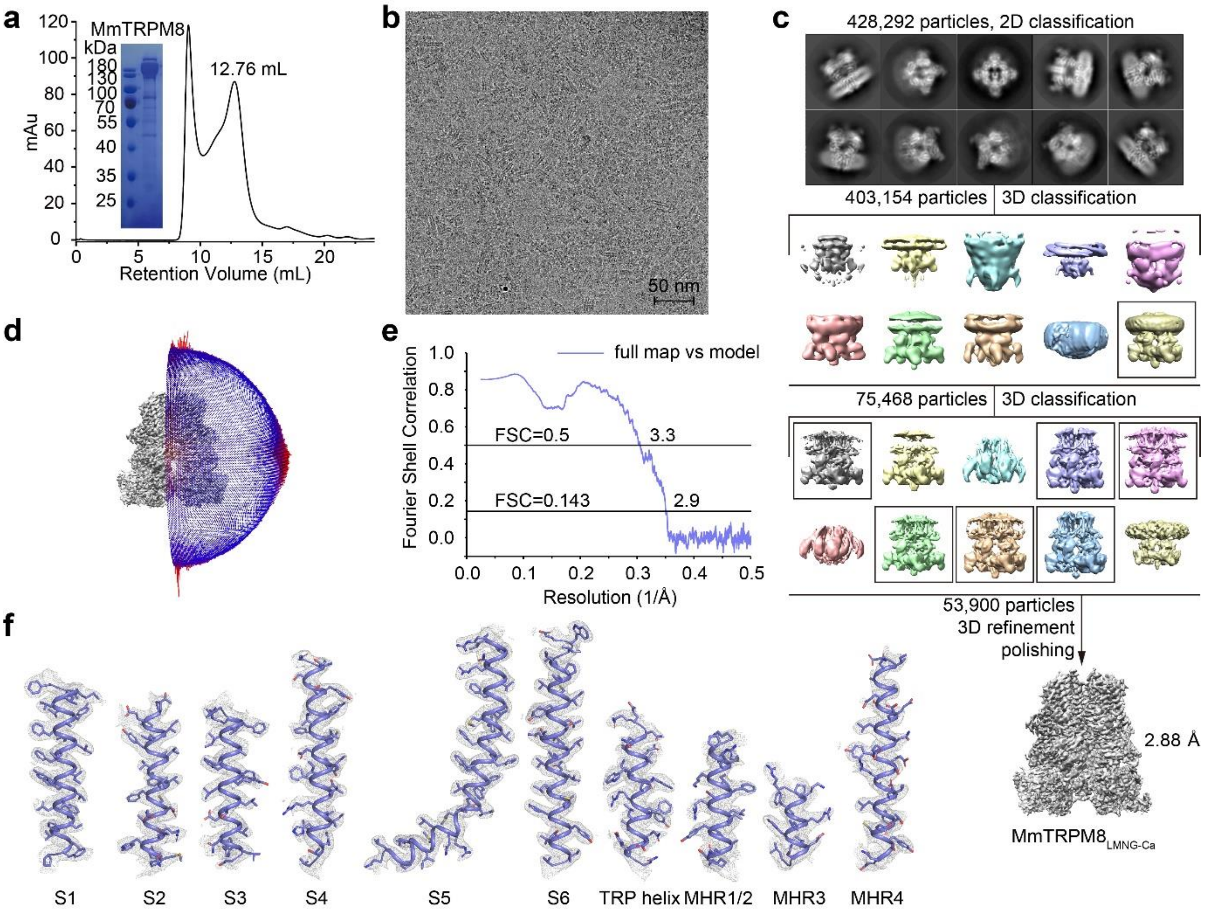

Supplementary Figure 4. Structure determination of MmTRPM8$_{LMNG\text{-}Ca}$. a, Size-exclusion chromatography of MmTRPM8$_{LMNG\text{-}Ca}$ on Superose 6 (GE Healthcare) and SDS-PAGE analysis of the final sample. n = 1 for SDS-PAGE analysis. **b**, Representative cryo-EM micrograph of MmTRPM8$_{LMNG\text{-}Ca}$. **c**, Flowchart of image processing for MmTRPM8$_{LMNG\text{-}Ca}$ particles. **d**, Angular distribution plot of particles included in the final *C4*-symmetric 3D reconstruction of MmTRPM8$_{LMNG\text{-}Ca}$. **e**, The FSC curves for cross-validation between the map and the model of MmTRPM8$_{LMNG\text{-}Ca}$. **f**, Sample maps at 10 helices of MmTRPM8$_{LMNG\text{-}Ca}$. For **a** and **e**, source data are provided as a Source Data file.

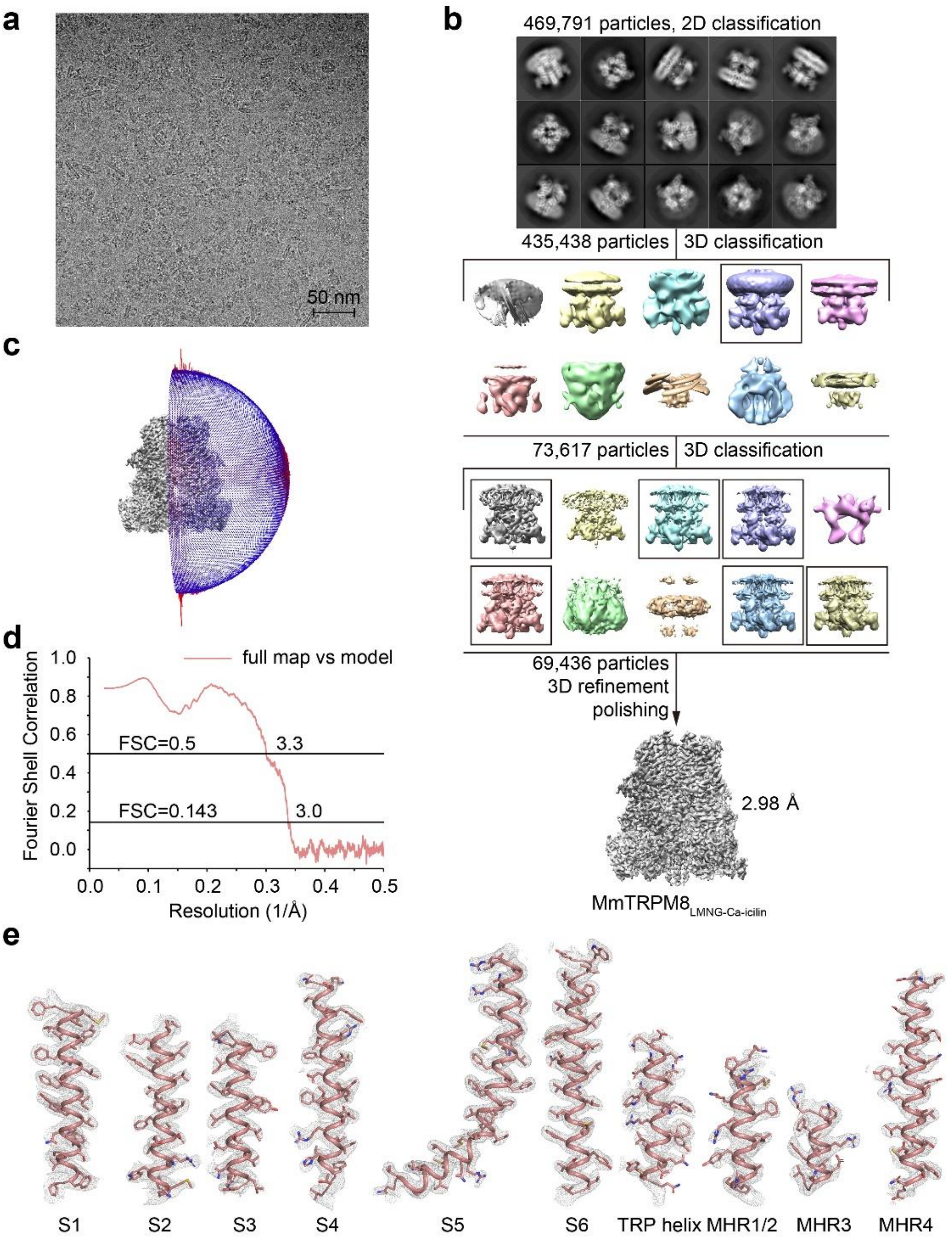

Supplementary Figure 5. Structure determination of MmTRPM8$_{\text{LMNG-Ca-icilin}}$. **a**, Representative cryo-EM micrograph of MmTRPM8$_{\text{LMNG-Ca-icilin}}$. **b**, Flowchart of image processing for MmTRPM8$_{\text{LMNG-Ca-icilin}}$ particles. **c**, Angular distribution plot of particles included in the final *C4*-symmetric 3D reconstruction of MmTRPM8$_{\text{LMNG-Ca-icilin}}$. **d**, The FSC curves for cross-validation between the map and the model of MmTRPM8$_{\text{LMNG-Ca-icilin}}$. Source data are provided as a Source Data file. **e,** Sample maps at 10 helices of MmTRPM8$_{\text{LMNG-Ca-icilin}}$.

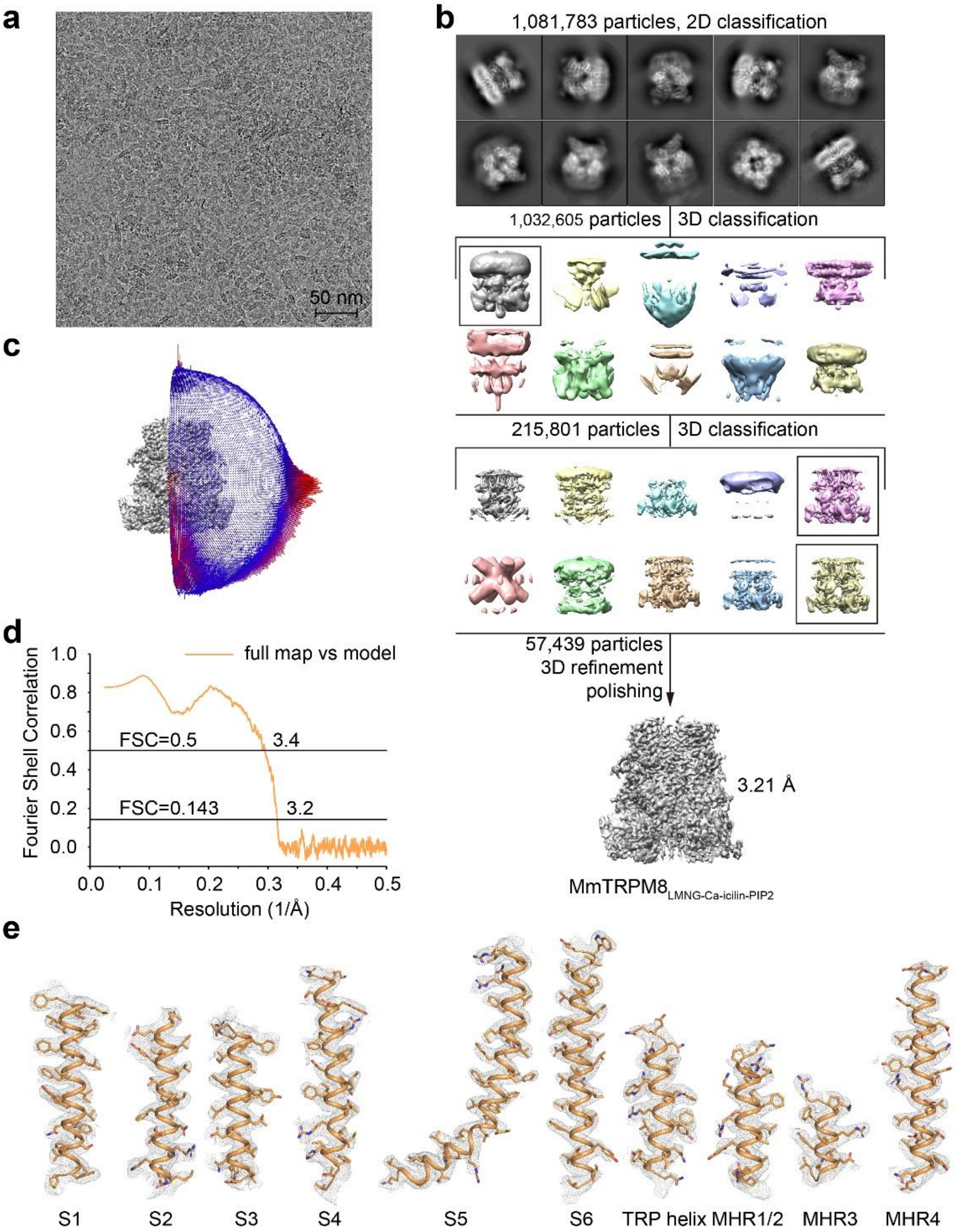

Supplementary Figure 6. Structure determination of MmTRPM8$_{\text{LMNG-Ca-icilin-PIP2}}$. a, Representative cryo-EM micrograph of MmTRPM8$_{\text{LMNG-Ca-icilin-PIP2}}$. **b**, Flowchart of image processing for MmTRPM8$_{\text{LMNG-Ca-icilin}}$ particles. **c**, Angular distribution plot of particles included in the final *C4*-symmetric 3D reconstruction of MmTRPM8$_{\text{LMNG-Ca-icilin-PIP2}}$. **d**, The FSC curves for cross-validation between the map and the model of MmTRPM8$_{\text{LMNG-Ca-icilin-PIP2}}$. Source data are provided as a Source Data file. **e,** Sample maps at 10 helices of MmTRPM8$_{\text{LMNG-Ca-icilin-PIP2}}$.

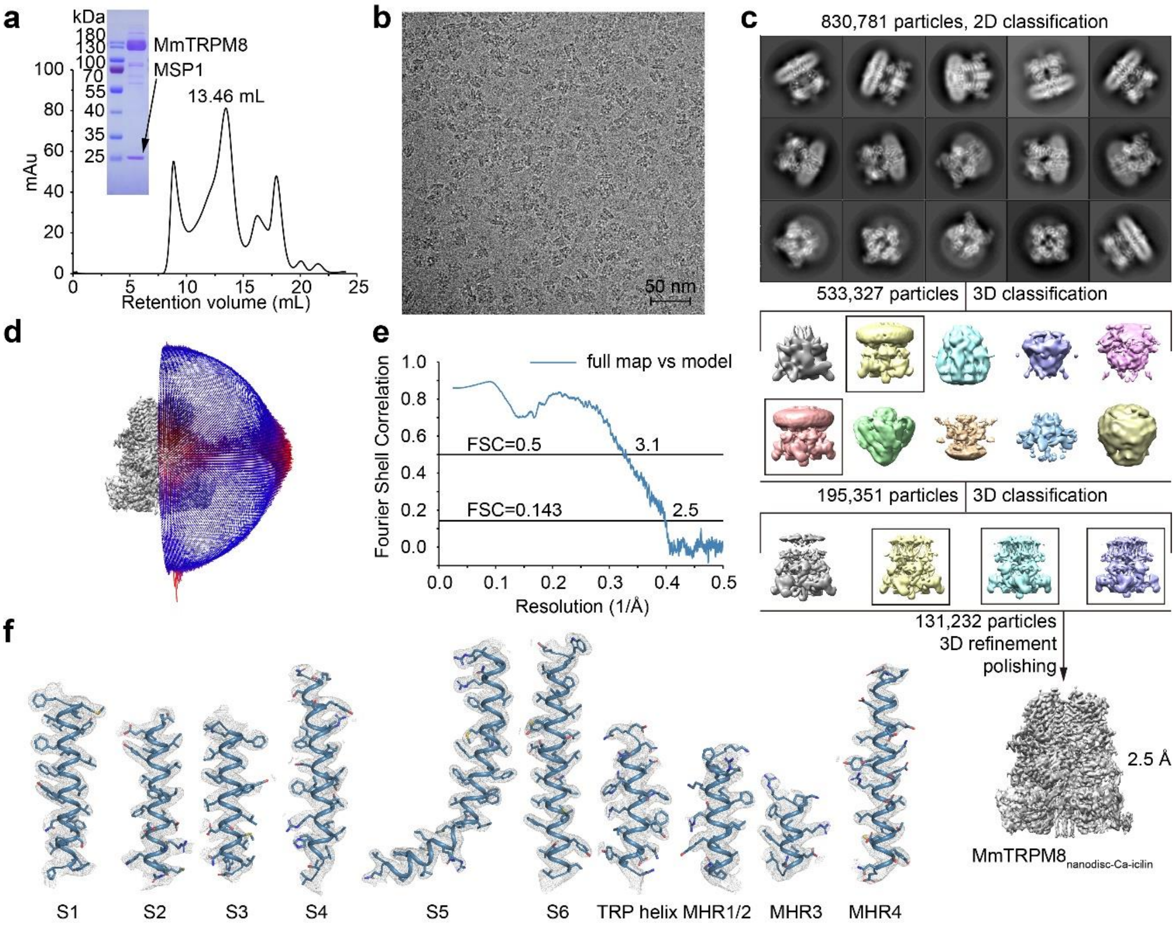

Supplementary Figure 7. Structure determination of MmTRPM8$_{\text{nanodisc-Ca-icilin}}$. a, Size-exclusion chromatography of MmTRPM8$_{\text{nanodisc-Ca-icilin}}$ on Superose 6 (GE Healthcare) and SDS-PAGE analysis of the final sample. n = 1 for SDS-PAGE analysis. **b**, Representative cryo-EM micrograph of MmTRPM8$_{\text{nanodisc-Ca-icilin}}$. **c**, Flowchart of image processing for MmTRPM8$_{\text{nanodisc-Ca-icilin}}$ particles. **d**, Angular distribution plot of particles included in the final *C4*-symmetric 3D reconstruction of MmTRPM8$_{\text{nanodisc-Ca-icilin}}$. **e**, The FSC curves for cross-validation between the map and the model of MmTRPM8$_{\text{nanodisc-Ca-icilin}}$. **f,** Sample maps at 10 helices of MmTRPM8$_{\text{nanodisc-Ca-icilin}}$. For **a** and **e**, source data are provided as a Source Data file.

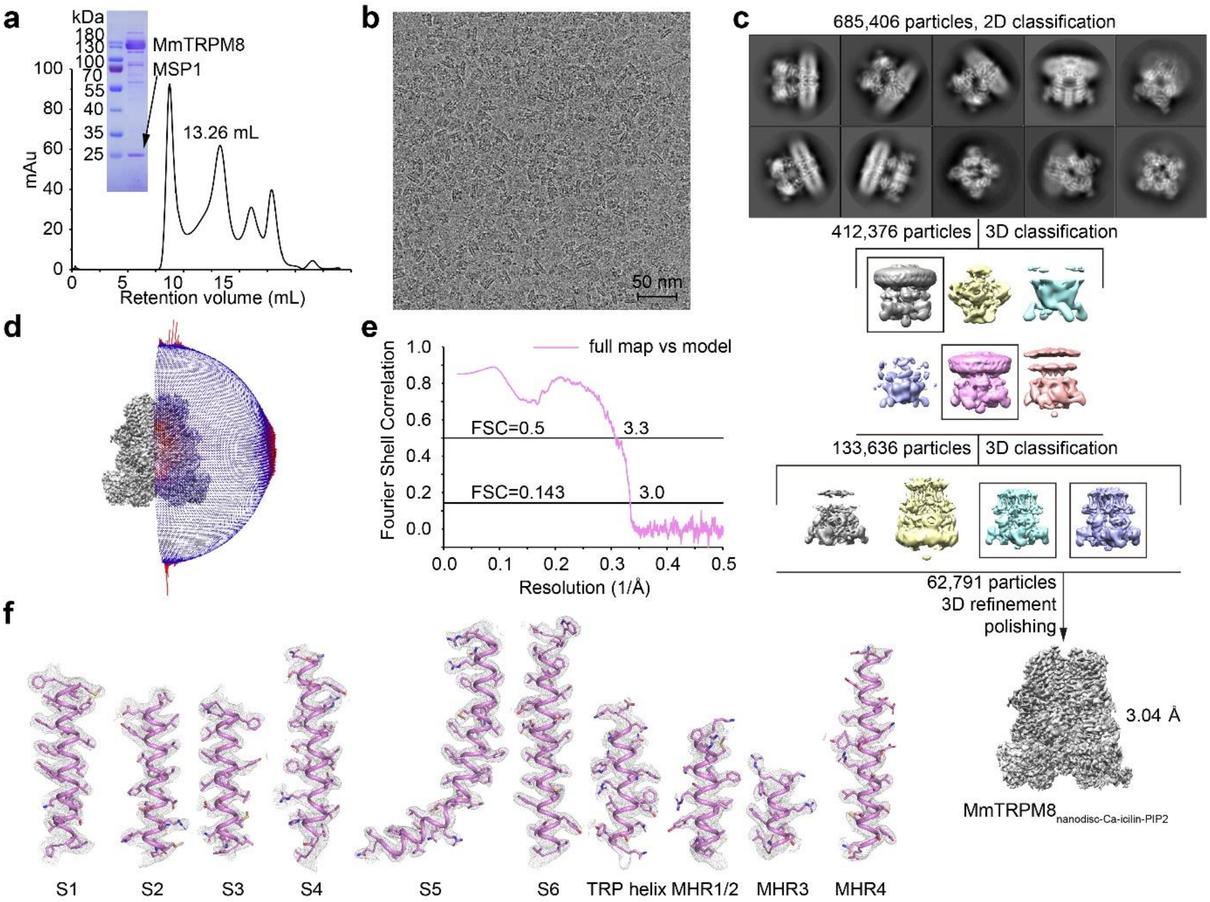

Supplementary Figure 8. Structure determination of MmTRPM8$_{\text{nanodisc-Ca-icilin-PIP2}}$. a, Size-exclusion chromatography of MmTRPM8$_{\text{nanodisc-Ca-icilin-PIP2}}$ on Superose 6 (GE Healthcare) and SDS-PAGE analysis of the final sample. n = 1 for SDS-PAGE analysis. **b**, Representative cryo-EM micrograph of MmTRPM8$_{\text{nanodisc-Ca-icilin-PIP2}}$. **c**, Flowchart of image processing for MmTRPM8$_{\text{nanodisc-Ca-icilin-PIP2}}$ particles. **d**, Angular distribution plot of particles included in the final *C4*-symmetric 3D reconstruction of MmTRPM8$_{\text{nanodisc-Ca-icilin-PIP2}}$. **e**, The FSC curves for cross-validation between the map and the model of MmTRPM8$_{\text{nanodisc-Ca-icilin-PIP2}}$. **f,** Sample maps at 10 helices of MmTRPM8$_{\text{nanodisc-Ca-icilin-PIP2}}$. For **a** and **e,** source data are provided as a Source Data file.

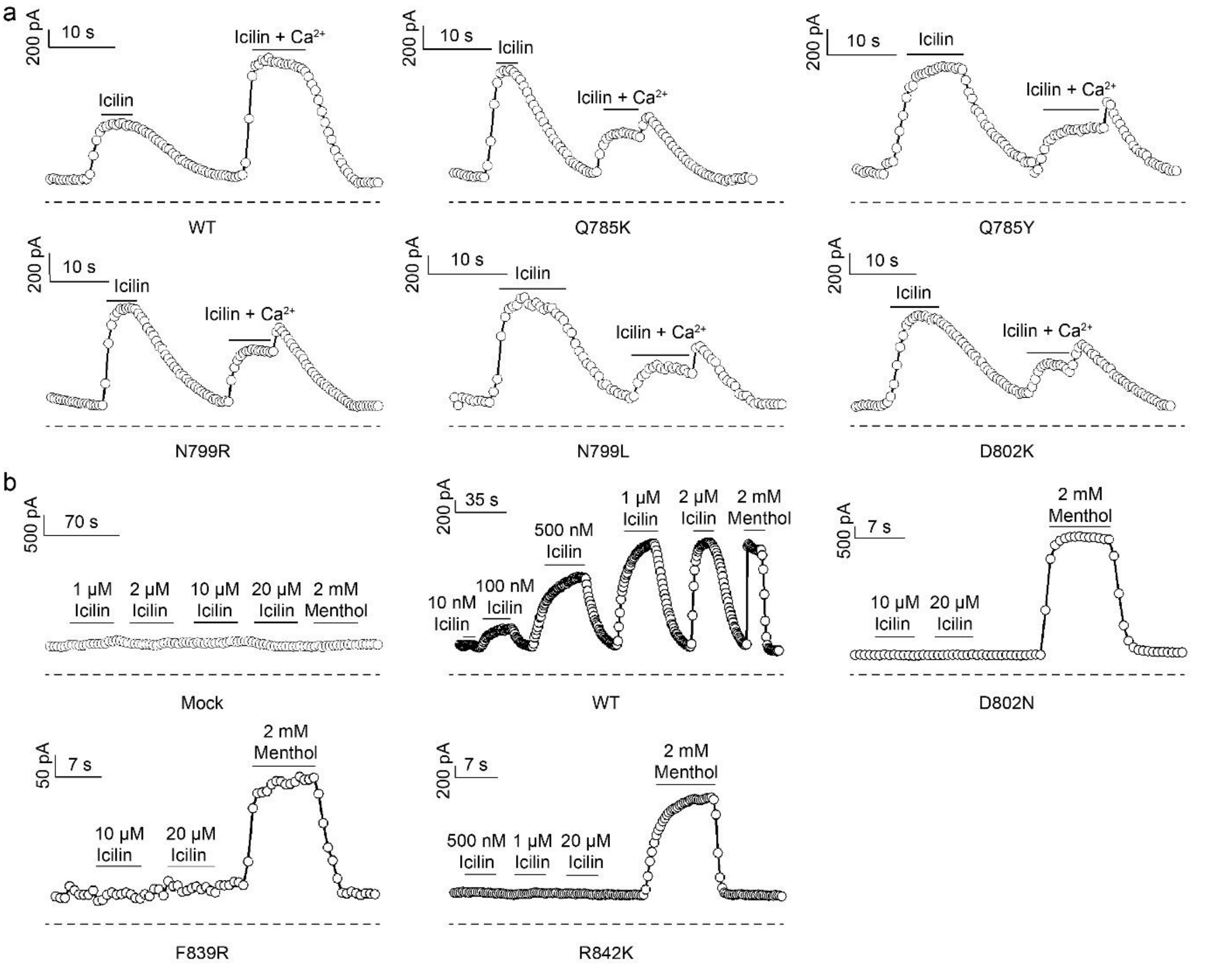

Supplementary Figure 9. The representative currents of WT and mutant MmTRPM8 in whole-cell patch-clamp recordings. a, The representative currents of WT and mutant MmTRPM8 activated by either icilin alone (10 μM) or in the presence of Ca^{2+} (1 mM) measured with whole-cell patch-clamp recordings. These Ca^{2+} binding site mutants show a reduced potentiation effect on the icilin activation of MmTRPM8. The dashed line indicated the baseline current. **b**, Representative ligand-induced whole-cell currents in HEK293 cells expressing empty vector (mock), WT, or mutant MmTRPM8. The three mutants D802N, F839R, and R842K are functional as they can be activated by 2 mM menthol.

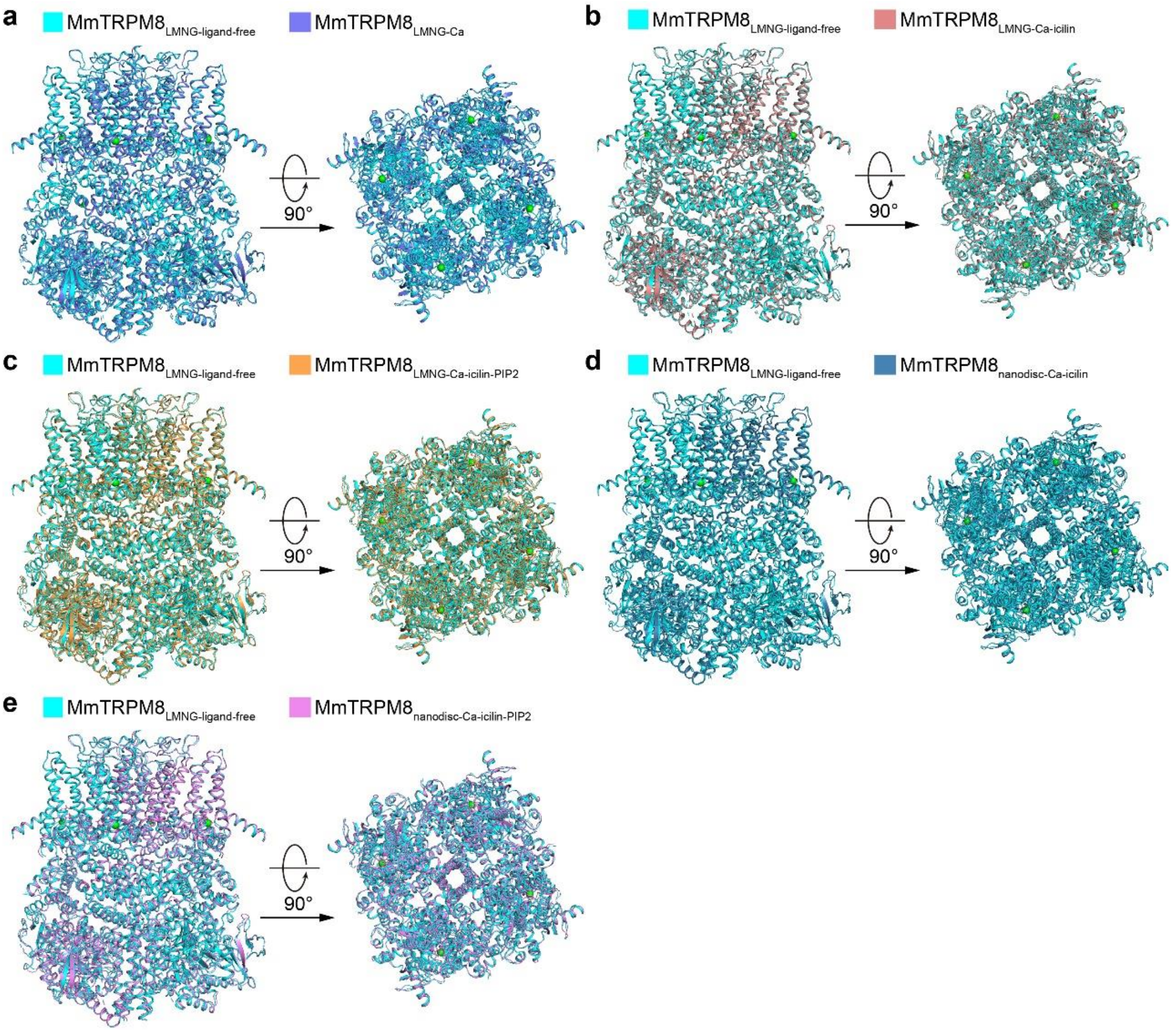

Supplementary Figure 10. Structure comparisons of MmTRPM8 in different ligand-bound states. a, Structural comparison of $MmTRPM8_{LMNG\text{-}ligand\text{-}free}$ and $MmTRPM8_{LMNG\text{-}Ca}$ when the entire channels are aligned. The RMSD over 930 Cα atoms within one subunit between two structures is 0.47 Å. **b,** Structural comparison of $MmTRPM8_{LMNG\text{-}ligand\text{-}free}$ and $MmTRPM8_{LMNG\text{-}Ca\text{-}icilin}$ when the entire channels are aligned. The RMSD over 930 Cα atoms within one subunit between two structures is 0.50 Å. **c,** Structural comparison of $MmTRPM8_{LMNG\text{-}ligand\text{-}free}$ and $MmTRPM8_{LMNG\text{-}Ca\text{-}icilin\text{-}PIP2}$ when the entire channels are aligned. The RMSD over 930 Cα atoms within one subunit between two structures is 0.75 Å. **d,** Structural comparison of $MmTRPM8_{LMNG\text{-}ligand\text{-}free}$ and $MmTRPM8_{nanodisc\text{-}Ca\text{-}icilin}$ when the entire channels are aligned. The RMSD over 930 Cα atoms within one subunit between two structures is 0.59 Å. **e,** Structural comparison of $MmTRPM8_{LMNG\text{-}ligand\text{-}free}$ and $MmTRPM8_{nanodisc\text{-}Ca\text{-}icilin\text{-}PIP2}$ when the entire channels are aligned. The RMSD over 930 Cα atoms within one subunit between two structures is 0.57 Å.

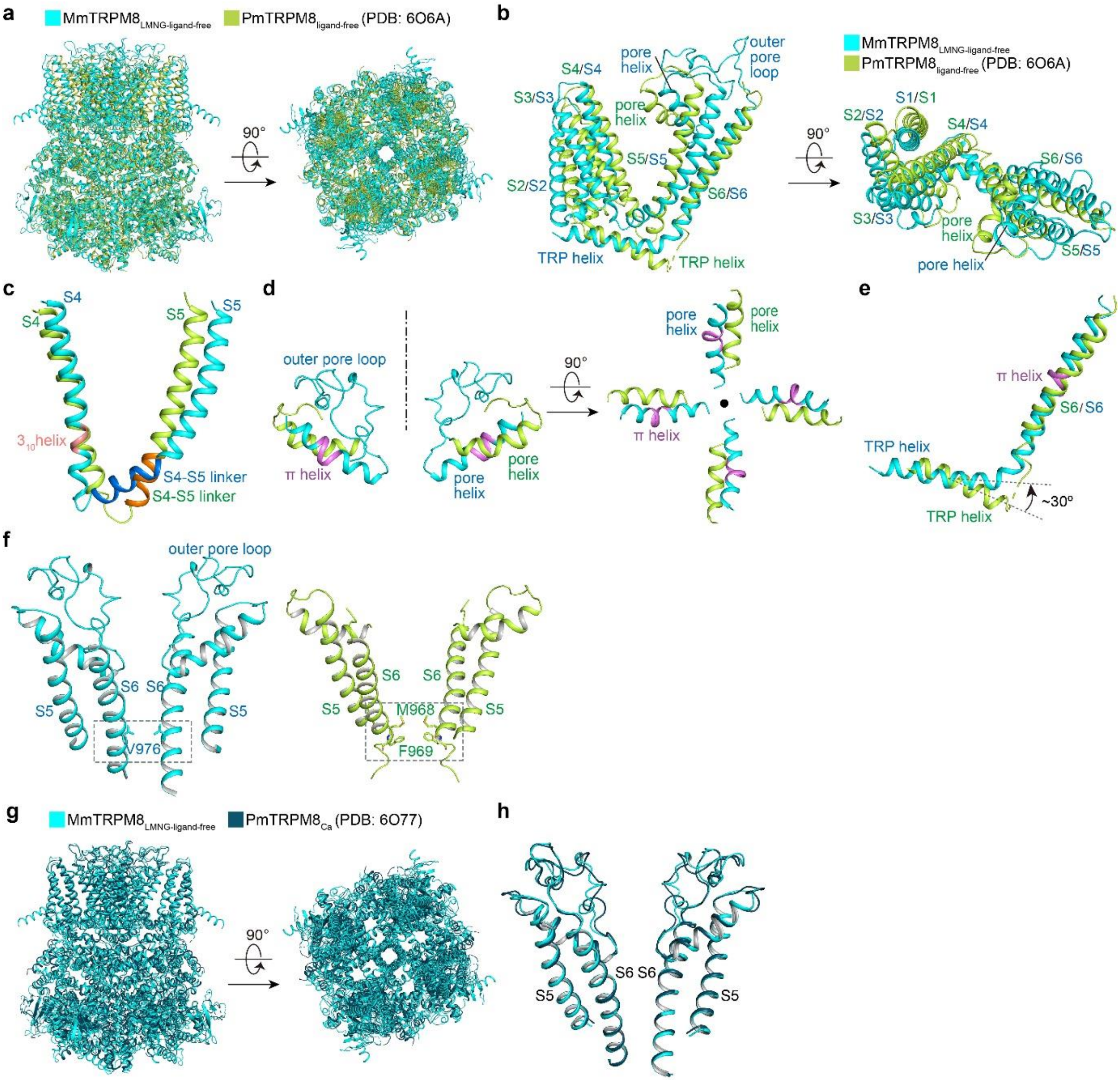

Supplementary Figure 11. Structural comparison of MmTRPM8$_{\text{ligand-free}}$ and PmTRPM8 in ligand-free (PmTRPM8$_{\text{ligand-free}}$, PDB: 6O6A) and Ca^{2+}-bound state (PmTRPM8$_{\text{Ca}}$, PDB: 6O77). a, Structural comparison of MmTRPM8$_{\text{LMNG-ligand-free}}$ and PmTRPM8$_{\text{ligand-free}}$ when the entire channels are aligned. **b**, Structural comparison of the S1–S6 in MmTRPM8$_{\text{LMNG-ligand-free}}$ and PmTRPM8$_{\text{ligand-free}}$. For clarity, only one subunit is shown. **c**, Structural comparison of S4 and S4-S5 linker in MmTRPM8$_{\text{LMNG-ligand-free}}$ and PmTRPM8$_{\text{ligand-free}}$. The 3_{10} helix in S4 of MmTRPM8$_{\text{LMNG-ligand-free}}$, and S4-S5 linker in two channels are highlighted. **d**, Structural comparison of pore helix, selectivity filter and outer pore loop in MmTRPM8$_{\text{LMNG-ligand-free}}$ and PmTRPM8$_{\text{ligand-free}}$. For clarity, the front and rear subunits in the side view, and the selectivity filter and outer pore loop in the top view are omitted. The π helix in the pore helix of MmTRPM8$_{\text{LMNG-ligand-free}}$ is highlighted. **e**, Structural comparison of S6 and

TRP helix in MmTRPM8$_{\text{LMNG-ligand-free}}$ and PmTRPM8$_{\text{ligand-free}}$. TRP helix in PmTRPM8$_{\text{ligand-free}}$ tilts by ~30 ° in comparison with that in MmTRPM8$_{\text{LMNG-ligand-free}}$. **f**, The activation gates of MmTRPM8$_{\text{LMNG-ligand-free}}$ and PmTRPM8$_{\text{ligand-free}}$. **g**, Structural comparison of MmTRPM8$_{\text{LMNG-ligand-free}}$ and PmTRPM8$_{\text{Ca}}$ when the entire channels are aligned. **h**, Structural comparison of the pore domain in MmTRPM8$_{\text{LMNG-ligand-free}}$ and PmTRPM8$_{\text{Ca}}$. For clarity, the front and rear subunits are omitted.

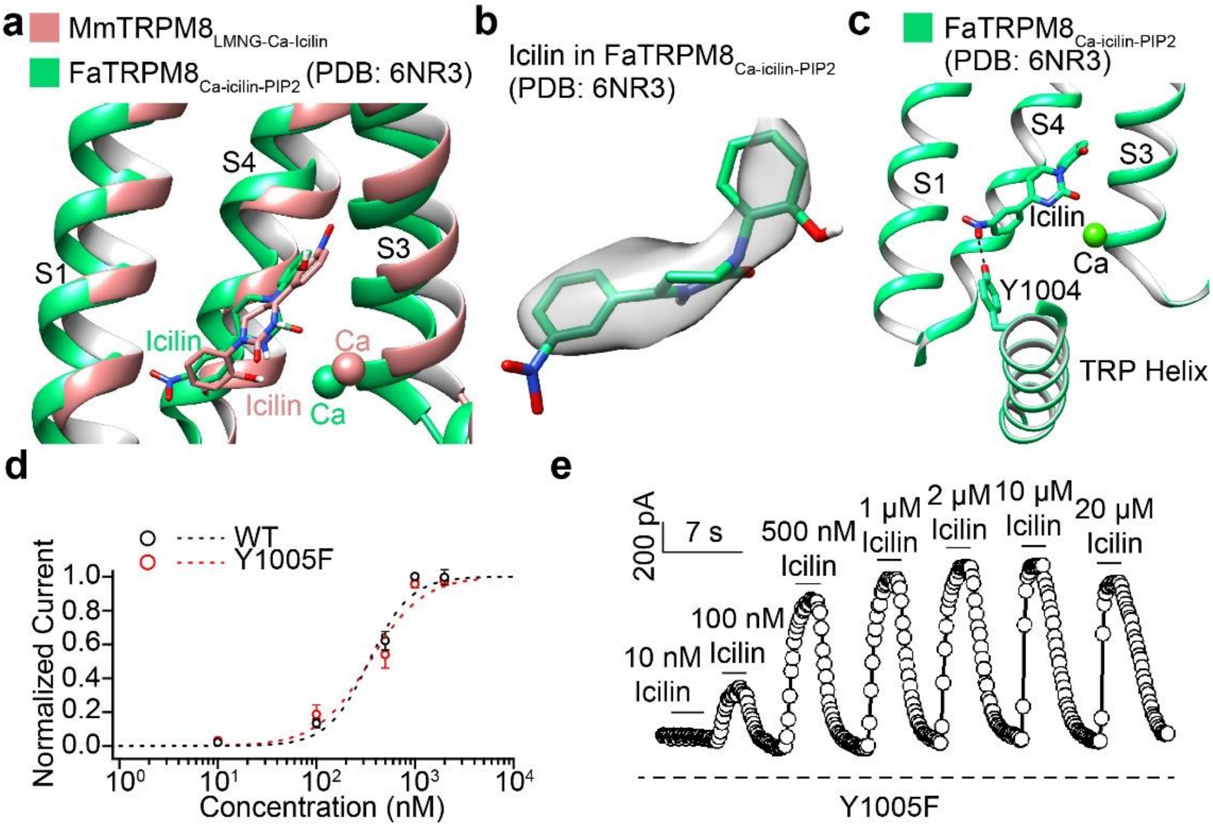

Supplementary Figure 12. The icilin binding sites in MmTRPM8$_{LMNG-Ca-icilin}$ and FaTRPM8$_{Ca-icilin-PIP2}$. **a**, Structural comparison of the icilin and Ca^{2+} binding site in MmTRPM8$_{LMNG-Ca-icilin}$ (salmon) and FaTRPM8$_{Ca-icilin-PIP2}$ (green, PDB: 6NR3). **b**, The icilin density in FaTRPM8$_{Ca-icilin-PIP2}$ (PDB: 6NR3) at the level of 0.032 in UCSF chimera. **c**, Icilin forms a strong hydrogen bond with Tyr1004 side chain in FaTRPM8$_{Ca-icilin-PIP2}$. **d**, Concentration-dependent icilin-activation shows that Y1005F mutant of MmTRPM8 (equivalent to Y1004F of FaTRPM8) maintains similar icilin sensitivity as the WT MmTRPM8 (For MmTRPM8, n = 5; for Y1005F, n = 3). All data points are mean ± s.e.m. Source data are provided as a Source Data file. **e**, Representative icilin-induced whole-cell currents in HEK293 cells expressing the MmTRPM8 Y1005F mutant.

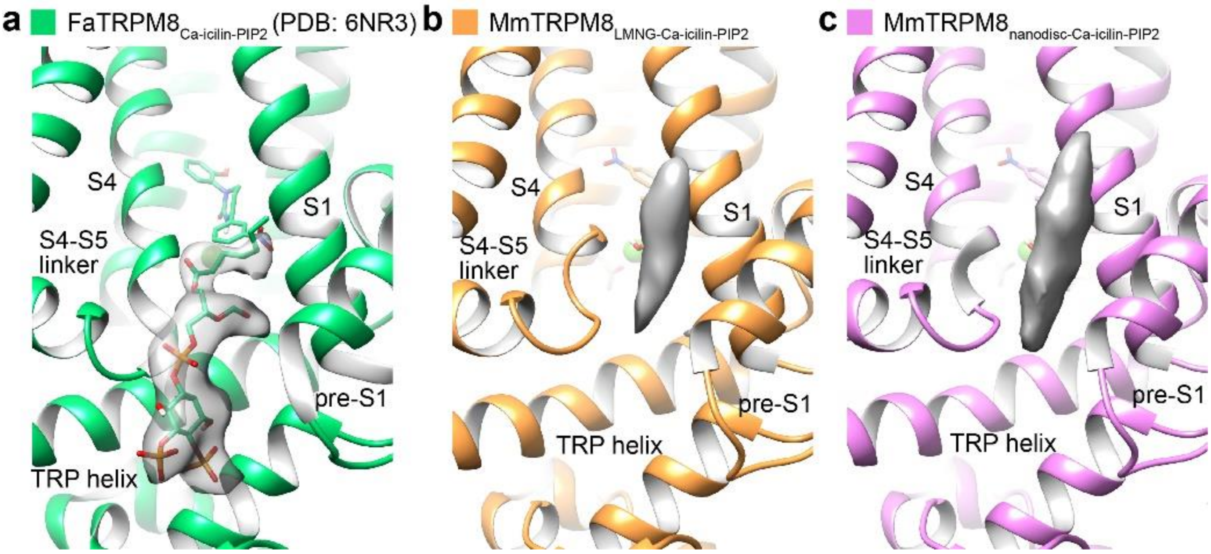

Supplementary Figure 13. The putative PIP_2 binding site in TRPM8. a, In $FaTRPM8_{Ca\text{-}icilin\text{-}PIP2}$ (PDB: 6NR3), PIP_2 binds at the cavity formed by the pre-S1 domain, S1, the junction of S4 and S5, and the TRP helix. The density of PIP2 is shown at the level of 0.2 in UCSF chimera. **b–c,** In the structures of $MmTRPM8_{LMNG\text{-}Ca\text{-}icilin\text{-}PIP2}$ (**b**) or $MmTRPM8_{nanodisc\text{-}Ca\text{-}icilin\text{-}PIP2}$ (**c**), bulk density corresponding to hydrophobic tails of lipid or detergent occupy the equivalent site of hydrophobic tails of PIP_2 in $FaTRPM8_{Ca\text{-}icilin\text{-}PIP2}$ at the level of 0.1 in UCSF chimera.

Supplementary Table 1. Data collection, model refinement, and validation statistics.

**Data collection and processing**	**MmTRPM8** **LMNG-ligand-free**	**MmTRPM8** **LMNG-Ca**	**MmTRPM8** **LMNG-Ca-icilin**	**MmTRPM8** **LMNG-Ca-icilin-PIP2**	**MmTRPM8** **nanodisc-Ca-icilin**	**MmTRPM8** **nanodisc-Ca-icilin-PIP2**
Microscope	FEI Titan Krios	FEI Titan Krios	FEI Titan Krios	FEI Titan Krios	FEI Titan Krios	FEI Titan Krios
Voltage (keV)	300	300	300	300	300	300
Camera	Gatan K2 Summit	Gatan K2 Summit	Gatan K2 Summit	Gatan K2 Summit	Gatan K2 Summit	Gatan K2 Summit
Magnification	49310×	49310×	49310×	49310×	49310×	49310×
Pixel size (Å)	1.014	1.014	1.014	1.014	1.014	1.014
Exposure rate (e−/pixel/sec)	8	8	8	8	8	8
Defocus range (μm)	−1.2 to −1.4	−1.2 to −1.4	−1.2 to −1.4	−1.2 to −1.4	−1.2 to −1.4	−1.2 to −1.4
Micrographs (no.)	1432	1884	2076	3082	1600	1602
Total extracted particles (no.)	324027	428292	469791	1081783	830781	685406
Refined particles (no.)	40653	53900	69436	57439	131232	62791
Map resolution (Å)	2.98	2.88	2.98	3.21	2.52	3.04
FSC threshold	0.143	0.143	0.143	0.143	0.143	0.143
**Refinement**						
Initial model (PDB code)	$MmTRPM8_{Ca}$	6O77	$MmTRPM8_{Ca}$	$MmTRPM8_{Ca}$	$MmTRPM8_{Ca}$	$MmTRPM8_{Ca}$
Model resolution (Å)	2.98	2.88	2.98	3.21	2.52	3.04
Map sharpening *B* factor (Å^2)	−40	−40	−60	−40	−10	−40
Model composition						
Non-hydrogen atoms	30373	30376	30468	30468	30468	30469
Protein residues	3720	3720	3720	3720	3720	3720
Ligands	1	4	8	8	8	9
B factor (Å^2)						
Protein	89.45	95.69	76.95	105.88	85.51	99.07
Ligand	26.44	78.43	41.41	79.22	54.82	69.10
r.m.s. deviation						
Bond lengths (Å^2)	0.007	0.006	0.009	0.01	0.012	0.008
Bond angles (°)	1.207	1.212	1.268	1.288	1.381	1.229
Validation						
MolProbity score	1.77	1.74	1.68	1.77	1.80	1.63
Clashscore	6.74	6.08	5.15	5.84	6.78	4.41
Rotamer outliers (%)	0.6	0.12	0.12	0.12	0.24	0.12
Ramachandran plot						
Favored (%)	94.04	93.87	93.98	93.00	93.52	93.82
Allowed (%)	5.96	6.02	5.80	7.00	6.15	6.18
Outliers (%)	0.00	0.11	0.22	0.00	0.33	0.00

Supplementary Table 2. Functional characterization of MmTRPM8 mutants. The function of these mutants was assessed by whole-cell patch-clamp recordings. Data shown as mean ± s.e.m.. n.d. indicates not determined.

Mutant	Icilin (20 µM) activation	Menthol (2 mM) activation	EC_{50} of icilin activation (nM)	Independent repeats
WT	Yes	Yes	401.0 ± 43.9	5
D802A	Yes	Yes	1403.6 ± 283.2	3
D802I	No	Yes	n.d.	5
D802L	No	Yes	n.d.	5
D802S	No	Yes	n.d.	5
D802N	No	Yes	n.d.	5
D802Q	No	Yes	n.d.	5
F839Y	Yes	Yes	2062.7 ± 418.1	3
F839R	No	Yes	n.d.	5
R842K	No	Yes	n.d.	5
R842N	No	Yes	n.d.	5
R842Q	No	Yes	n.d.	5
Y1005F	Yes	Yes	458.4 ± 52.7	5
