# Supplementary material for: Structures of a mammalian TRPM8 in closed state
Source: Nat Commun. 2022 Jun 3;13:3113. doi: 10.1038/s41467-022-30919-y (PMC9166780; doi:10.1038/s41467-022-30919-y)
Supplement: Supplementary file 3 — Reporting Summary [file 41467_2022_30919_MOESM3_ESM.pdf]

nature portfolio

Corresponding author(s): Jiangtao Guo, Fan Yang, Yuan Xie

Last updated by author(s): May 17, 2022

Reporting Summary

Nature Portfolio wishes to improve the reproducibility of the work that we publish. This form provides structure for consistency and transparency in reporting. For further information on Nature Portfolio policies, see our Editorial Policies and the Editorial Policy Checklist.

Statistics

For all statistical analyses, confirm that the following items are present in the figure legend, table legend, main text, or Methods section.

n/a	Confirmed	
☐	☒	The exact sample size (*n*) for each experimental group/condition, given as a discrete number and unit of measurement
☐	☒	A statement on whether measurements were taken from distinct samples or whether the same sample was measured repeatedly
☐	☒	The statistical test(s) used AND whether they are one- or two-sided<br>*Only common tests should be described solely by name; describe more complex techniques in the Methods section.*
☒	☐	A description of all covariates tested
☒	☐	A description of any assumptions or corrections, such as tests of normality and adjustment for multiple comparisons
☐	☒	A full description of the statistical parameters including central tendency (e.g. means) or other basic estimates (e.g. regression coefficient) AND variation (e.g. standard deviation) or associated estimates of uncertainty (e.g. confidence intervals)
☐	☒	For null hypothesis testing, the test statistic (e.g. *F*, *t*, *r*) with confidence intervals, effect sizes, degrees of freedom and *P* value noted<br>*Give P values as exact values whenever suitable.*
☒	☐	For Bayesian analysis, information on the choice of priors and Markov chain Monte Carlo settings
☒	☐	For hierarchical and complex designs, identification of the appropriate level for tests and full reporting of outcomes
☒	☐	Estimates of effect sizes (e.g. Cohen's *d*, Pearson's *r*), indicating how they were calculated

Our web collection on statistics for biologists contains articles on many of the points above.

Software and code

Policy information about availability of computer code

Data collection	SerialEM (v 3.8)
Data analysis	RELION (v 3.0); Coot (v 0.8.9.1); UCSF Chimera (v 1.13.1); ; Phenix (v 1.15.2-3472); MotionCor2 (v 1.1.0); Gctf (v 1.06); PyMOL (v 2.6.0)

For manuscripts utilizing custom algorithms or software that are central to the research but not yet described in published literature, software must be made available to editors and reviewers. We strongly encourage code deposition in a community repository (e.g. GitHub). See the Nature Portfolio guidelines for submitting code & software for further information.

Data

Policy information about availability of data

All manuscripts must include a data availability statement. This statement should provide the following information, where applicable:
- Accession codes, unique identifiers, or web links for publicly available datasets
- A description of any restrictions on data availability
- For clinical datasets or third party data, please ensure that the statement adheres to our policy

The cryo-EM density maps have been in the Electron Microscopy Data Bank (EMDB) under accession numbers EMD-32720 [https://www.ebi.ac.uk/emdb/EMD-32720] (MmTRPM8LMNG-ligand-free), EMD-32721 [https://www.ebi.ac.uk/emdb/EMD-32721] (MmTRPM8LMNG-Ca), EMD-32723 [https://www.ebi.ac.uk/emdb/EMD-32723] (MmTRPM8LMNG-Ca-icilin), EMD-32722 [https://www.ebi.ac.uk/emdb/EMD-32722] (MmTRPM8LMNG-Ca-icilin-PIP2), EMD-32724 [https://www.ebi.ac.uk/emdb/EMD-32724] (MmTRPM8nanodisc-Ca-icilin), and EMD-32725 [https://www.ebi.ac.uk/emdb/EMD-32725] (MmTRPM8nanodisc-Ca-icilin-PIP2). The coordinates have been in the RCSB Protein Data Bank (PDB) under accession codes 7WRA [https://www.rcsb.org/structure/7WRA] (MmTRPM8LMNG-ligand-free), 7WRB [https://www.rcsb.org/structure/7WRB] (MmTRPM8LMNG-Ca), 7WRD [https://www.rcsb.org/structure/7WRD] (MmTRPM8LMNG-Ca-icilin), 7WRC [https://www.rcsb.org/structure/7WRC] (MmTRPM8LMNG-Ca-icilin-PIP2), 7WRE [https://www.rcsb.org/structure/7WRE] (MmTRPM8nanodisc-Ca-icilin), and 7WRF

[https://www.rcsb.org/structure/7WRF] (MmTRPM8nanodisc-Ca-icilin-PIP2).

Field-specific reporting

Please select the one below that is the best fit for your research. If you are not sure, read the appropriate sections before making your selection.

- [x] Life sciences
- [ ] Behavioural & social sciences
- [ ] Ecological, evolutionary & environmental sciences

For a reference copy of the document with all sections, see nature.com/documents/nr-reporting-summary-flat.pdf

Life sciences study design

All studies must disclose on these points even when the disclosure is negative.

Sample size	No statistical methods were used to predetermine sample size. The data size for cryoEM was determined by the availability of the microscope time and the particle density on the grids. For the electrophysiology assays, experiments were performed at least three independent times. The sample size was determined based on the reproducibility of the current recording.
Data exclusions	CryoEM data processing involved removing poor-quality or damaged particles to achieve high resolution maps through pre-established standard data classification procedures.
Replication	Each experiment was repeated at least three times in independent experiments. Experimental findings were reproduced reliably.
Randomization	For structure refinement, all particles were randomly split into two groups, one group was used for refinement and the other for validation. For the eletrophysiology assays, randomization is not relevant as no group allocation were performed.
Blinding	Investigators were not blinded to group allocation, because no grouping was needed for this study.

Reporting for specific materials, systems and methods

We require information from authors about some types of materials, experimental systems and methods used in many studies. Here, indicate whether each material, system or method listed is relevant to your study. If you are not sure if a list item applies to your research, read the appropriate section before selecting a response.

Materials & experimental systems

n/a	Involved in the study	
x		Antibodies
	x	Eukaryotic cell lines
x		Palaeontology and archaeology
x		Animals and other organisms
x		Human research participants
x		Clinical data
x		Dual use research of concern

Methods

n/a	Involved in the study	
x		ChIP-seq
x		Flow cytometry
x		MRI-based neuroimaging

Eukaryotic cell lines

Policy information about cell lines

Cell line source(s)	HEK293F: Thermo Fisher Scientific; R79007<br>HEK293T: ATCC; CRL-3216<br>Sf9: ATCC; CRL-1711
Authentication	No further authentications were performed for this study.
Mycoplasma contamination	No mycoplasma contamination tests were performed for this study.
Commonly misidentified lines (See ICLAC register)	None of the cell lines used are listed in the ICLAC database.
